# Author Correction: Self-assembly of Co/Pt stripes with current-induced domain wall motion towards 3D racetrack devices

**DOI:** 10.1038/s41467-024-47944-8

**Published:** 2024-04-26

**Authors:** Pavel Fedorov, Ivan Soldatov, Volker Neu, Rudolf Schäfer, Oliver G. Schmidt, Daniil Karnaushenko

**Affiliations:** 1 Research Center for Materials, Architectures and Integration of Nanomembranes (MAIN), Chemnitz University of Technology, 09126 Chemnitz, Germany; 2 Leibniz Institute for Solid State and Materials Research, 01069 Dresden, Germany; 3 Institute for Materials Science, TU Dresden, 01062 Dresden, Germany; 4 Material Systems for Nanoelectronics, Chemnitz University of Technology, 09107 Chemnitz, Germany; 5 Nanophysics, Faculty of Physics, TU Dresden, 01062 Dresden, Germany

**Keywords:** Electrical and electronic engineering, Magnetic properties and materials

Correction to: *Nature Communications* 10.1038/s41467-024-46185-z, published online 06 March 2024

The original version of this article contained an error in Fig. 1a, in which a label was misaligned. The correct version of Fig. 1a is:
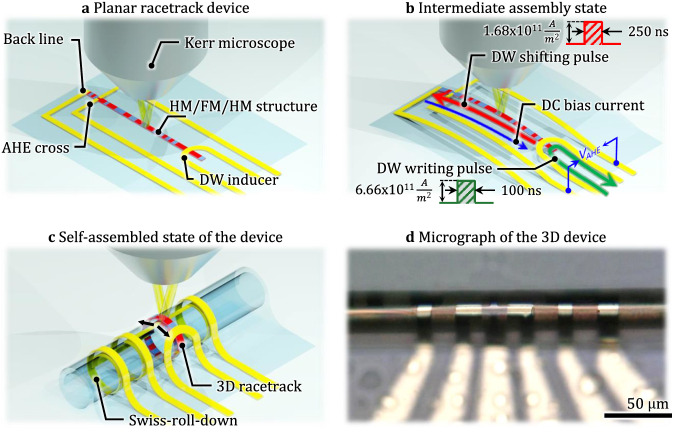


which replaces the previous incorrect version:
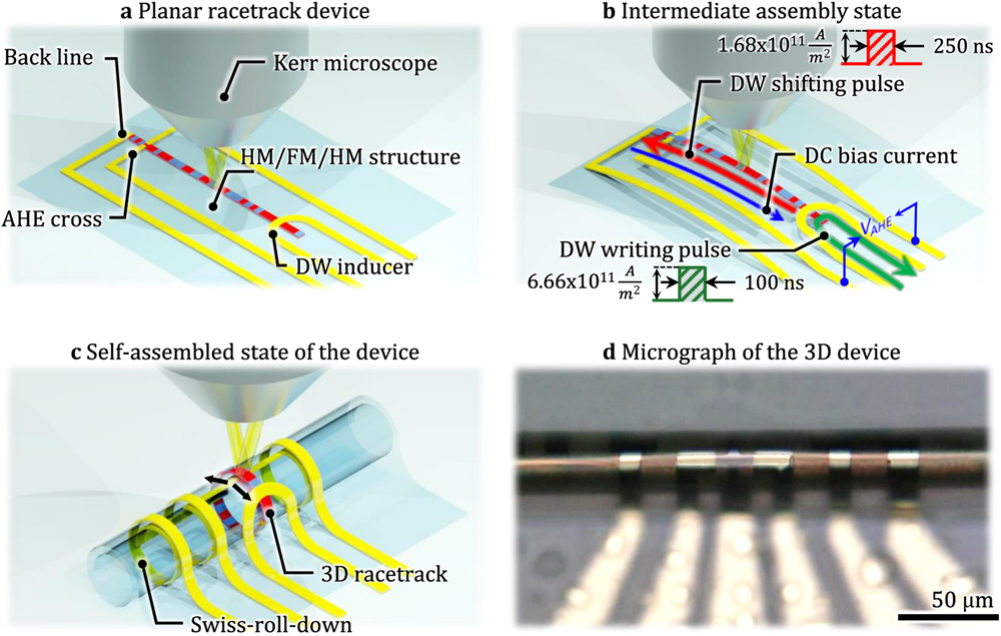


This has been corrected in both the PDF and HTML versions of the Article.

The original version of this article contained an error in Fig. 3f, in which a label was not readable. The correct version of Fig. 3f is:
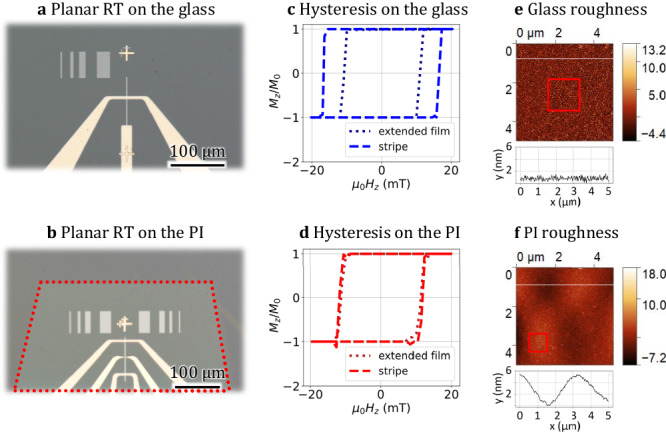


which replaces the previous incorrect version:
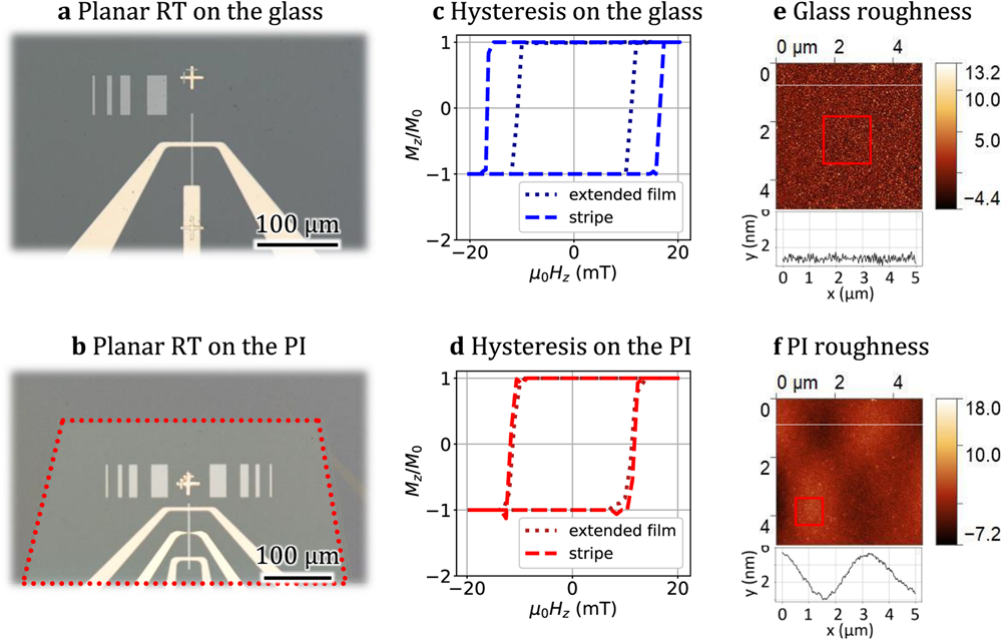


This has been corrected in both the PDF and HTML versions of the Article.

