# Supplementary material for: Self-assembly of Co/Pt stripes with current-induced domain wall motion towards 3D racetrack devices
Source: Nat Commun. 2024 Mar 6;15:2048. doi: 10.1038/s41467-024-46185-z (PMC10918081; doi:10.1038/s41467-024-46185-z)
Supplement: Supplementary file 1 — Supplementary Information [file 41467_2024_46185_MOESM1_ESM.pdf]

Supplementary information

Self-assembly of Co/Pt stripes with current-induced domain wall motion towards 3D racetrack devices.

Pavel Fedorov et al.

TABLE OF CONTENTS

Supplementary Figures

Supplementary figure 1: Schematic illustration of the neutral axis and the cross-section of the rolled-down polymer tube. a in a planar state, and b after rolling down.

Supplementary figure 2: Hysteresis loop measurement with Hx = 0 mT, that confirms a proper alignment of the Hall cross and the Hz field for the SOT measurement in the rolled configuration.

Supplementary figure 3: Kerr images of current-induced motion of domain wall in 3D tubular geometry.

Supplementary Tables

Supplementary table 1: Coercivity change after the RT was self-assembled from the 2D to 3D state.

Supplementary Notes

Supplementary note 1. Strain estimation of rolled geometries

Supplementary Figures

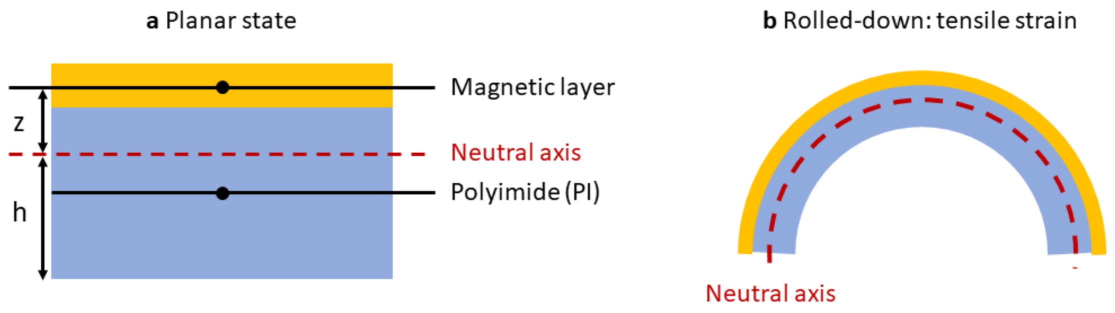

Supplementary figure 1: Schematic illustration of the neutral axis and the cross-section of the rolled-down polymer tube**. a** in a planar state, and **b** after rolling down.

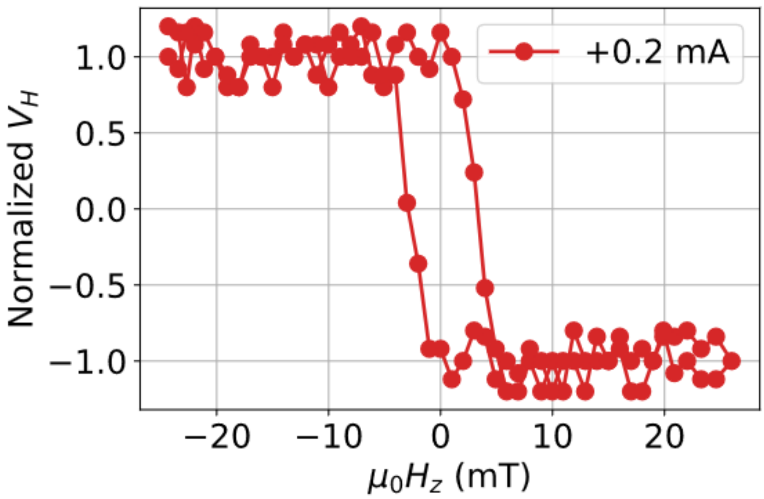

Supplementary figure 2: Hysteresis loop measurement with Hx = 0 mT, that confirms a proper alignment of the Hall cross and the Hz field for the SOT measurement in the rolled configuration.

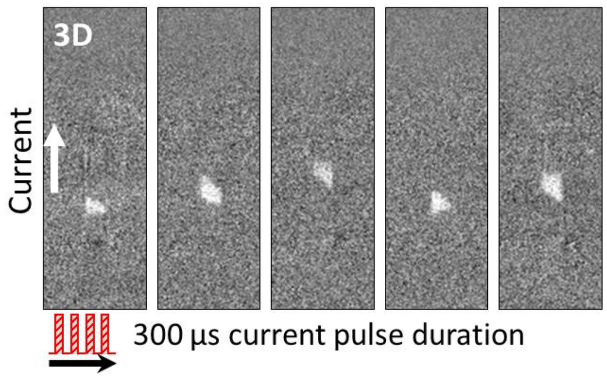

Supplementary figure 3: Kerr images of current-induced motion of domain wall in 3D tubular geometry.

Supplementary Tables

	2D (mT)	3D (mT)
Device #1	8.58	9.85
Device #1	8.83	10.62

Supplementary table 1: Coercivity change after the RT was self-assembled from the 2D to 3D state.

Supplementary notes

Supplementary note 1. Strain estimation of rolled geometries

Here we demonstrate the strain calculation for the tubes, used for the 3D DW motion. Supplementary figure 1 illustrates the cross-section of the layered structure: polyimide layer with functional magnetic thin film on the top together with the neutral axis. The neutral axis is an axis in the cross-section where there is no bending strain over the bilayer.

We calculate the strain for rolled down tubes in the main text. First, we calculate the biaxial modulus:

$$B = E/(1-v)$$

where E - Young's modulus, v - Poisson's ratio. The thickness of Polyimide film and RT stripe are 600 nm and 10 nm, respectively. $B_{PI} = E_{PI}/(1-v_{PI}) = 6.4*10^9$ and $B_{RT} = E_{RT}/(1-v_{RT}) = 1.71*10^{11}$, with $E_{PI(RT)} = 3.2*10^9$ $(1.06*10^{11})$ and $v_{PI(RT)} = 0.5$ (0.38). The neutral axis estimated as:

$$h_{NA} = \frac{B_{PI}h_{PI}^2 + B_{RT}(h_{RT}^2 + 2h_{PI}h_{RT})}{2(B_{PI}h_{PI} + B_{RT}h_{RT})} = 394 \text{ nm}$$

This means, the neutral axis is 394 nm from the bottom of PI layer. The distance from the neutral axis to the middle of the RT stripe is 211 nm.

The tensile strain is then estimated from:

$$\varepsilon=z/R$$

where R is the radius of the tube, 16 µm. This results in ε = 0.0131, that is 1.31% of the compressive strain for the rolled-down tube.
